# Chinese mental health workers’ family-focused practices: a cross-sectional survey

**DOI:** 10.1186/s12913-021-06572-4

**Published:** 2021-06-09

**Authors:** Hao Yao, Lili Guan, Changchun Zhang, Yang Pan, Jinxiang Han, Rui He, Zhengjiao Chang, Tianhang Zhou, Chunyu Du, Tingfang Wu, Jingwen Sun, Yilin Yuan, Darryl Maybery, Hong Ma

**Affiliations:** 1 Harvard T. H. Chan School of Public Health, Boston, MA USA; 2 Shanghai Clinical Research Center for Mental Health, Shanghai Key Laboratory of Psychotic Disorders, Shanghai Mental Health Center, Shanghai Jiao Tong University School of Medicine, Shanghai, China; 3 Peking University Sixth Hospital, Peking University Institute of Mental Health, NHC Key Laboratory of Mental Health (Peking University), National Clinical Research Center for Mental Disorders (Peking University Sixth Hospital), Beijing, China; 4 Fangshan District Mental Health Care Hospital, Beijing, China; 5 Beijing Xicheng District Ping’an Hospital, Beijing, China; 6 Mental Health Prevention Hospital of Haidian District, Beijing, China; 7 Department of Rural Health, Faculty of Medicine, Nursing and Health Sciences, Monash University, 15 Sargeant Street, 3820 Warragul, Victoria Australia

**Keywords:** Mental health services, Family-focused practices, Children of parents with mental illness, China, Parenting

## Abstract

**Background:**

Mental disorders impose heavy burdens on patients’ families and children. It is imperative to provide family-focused services to avoid adverse effects from mental disorders on patients’ families and children. However, implementing such services requires a great deal of involvement of mental health workers. This study investigated the attitudes, knowledge, skills, and practices in respect to family-focused practices (FFP) in a sample of Chinese mental health workers.

**Methods:**

A cross-sectional study design was employed to examine the attitudes, knowledge, skills, and practices of a convenience sample of Chinese mental health workers in respect to FFP, using the Chinese version of the Family-Focused Mental Health Practice Questionnaire (FFMHPQ).

**Results:**

In total, 515 mental health workers participated in our study, including 213 psychiatrists, 269 psychiatric nurses, and 34 allied mental health professionals (20 clinical psychologists, 9 mental health social workers, and 4 occupational therapists). Compared with psychiatric nurses, psychiatrists and allied mental health professionals provided more support for families and children of patients with mental illness and were more willing to receive further training in FFP. However, there were no significant differences on knowledge, skills, and confidence across different profession types. After adjusting for demographic and occupational variables, previous training in FFP was positively associated with mental health workers’ knowledge, skills, and confidence about FFP, but not actual support to families and children.

**Conclusions:**

Professional differences on FFP exist in Chinese mental health workers. Training is needed to engage psychiatrists and other allied workforce in dissemination and implementation of FFP in China.

## Βackground

Globally, mental illness is recognized as an important and severe public health problem. It is estimated that more than one billion people worldwide are afflicted with mental illness, making up 16 % of the world’s population [1]. Mental illness is a major cause of non-fatal burden in the world, accounting for 18.7 % of years of lived with disability in 2016 [2]. In China, the burden attributable to mental disorders has increased by 28 % over the last twenty years [3]. The prevalence of mental disorders (excluding dementia) among adults in China has risen to 9.3 % in 2013 [4], much higher than in 1982 (point prevalence 1.1 %) [5].

 Mental disorders, especially those in the most severe forms, impose heavy burdens on patients’ families and, in particular, on children of parents with mental illness (COPMI). It has been reported that up to one in five children and adolescents lived in households where at least one parent has mental illness [6]. Many factors, including genetics [7], interrupted family dynamics [8], deprivation of love and care from parents with mental illness [9], and social stigma [10], can put COPMI at significantly higher risks of mental disorders than other children. It was estimated that 41–77 % COPMI were likely to develop mental disorders [11]. Fortunately, some of these contributing factors can be alleviated and even be eliminated if COPMI are identified and supported by mental health workers as early as possible [9]. Of great importance is that several recent systematic reviews have shown that 20–38.5 % of people attending adult psychiatric services were parents [12] and the prevalence of mental illness among parents of children receiving treatment within child and adolescent mental health services ranged from 16 to 79 % [13]. The most accurate study highlighted that 36 % of mothers and 33 % of fathers of children and adolescents attending psychiatric services were also suffering from mental illness [13]. Thus, it is clear that a family-focused approach in psychiatry that goes beyond working exclusively with patients and addresses the needs of their families, particularly those of their children, is needed. Family-focused practice (FFP) is a commonly used term to describe such a holistic approach [14]. In recent decades, many studies have indicated that family-focused mental health services, including family-intervention programs, peer support programs, online interventions, and bibliotherapy [15] can decrease the risk of developing mental disorders in COPMI by 40 % [16].

It is notable that more and more governments have introduced relevant policies to advocate for a family-focused model of mental health services. For instance, the Norwegian government amended their health legislation (the *Health Personal Act*) in 2010 [17] to require that all health workers should identify and support dependent children of their clients, including those with mental disorders [18]. Canada also issued many government documents (e.g., *Rising to the Challenge: A Strategic Plan for the Mental Health and Well-being for Manitobans* [19]) for the recognition and support of families of patients with mental disorders. Recently, in China, the National Health Commission published its first ever *Action Plan for Child and Adolescent Mental Health* [20] where it was also explicitly required that family-focused preventive interventions should be provided to COPMI in order to prevent intergenerational transmission of mental disorders.

Despite the introduction of these national policies to advocate for family-focused services, there is no guarantee that they will be effectively implemented as expected. For example, in Norway, Lauritzen et al. found that the incorporation of FFP into mental health services was slow since the 2010 amendment of the *Health Personal Act* [21]. Nicholson also reported that, in Canada, COPMI were still not typically identified and supported in spite of relevant government policies [22]. In order for a family-focused model to be integrated into the existing mental health services at local or national level, mental health workers should be, first of all, proactively involved in FFP, as well as being equipped with adequate knowledge and skills to adopt a family-focused approach. Many studies, however, revealed that, although mental health workers acknowledged the importance of working with COPMI, many of them were faced with a deficiency of knowledge and skills to work with patients’ families, which constituted a prominent obstacle for them to integrate FFP into mental health services [17, 23–26]. Although the family-focused perspective was incorporated into the *China National Action Plan for Child and Adolescent Mental Health* [20], we are still unaware what attitudes Chinese mental health workers hold towards FFP and whether they consider themselves to be well prepared to work with COPMI in clinical practices. Moreover, prior research found that different types of mental health workers varied on their attitudes, knowledge, skills, and practices in respect to FFP [14, 27]. For example, in Australia, psychiatric nurses engaged less in FFP compared to psychologists, and both less than mental health social workers [27]. In Thailand, it was reported that psychiatric nurses were less confident of and less skilled at providing FFP to patients’ families than other mental health professionals [14]. Therefore, it is also essential to make comparisons on FFP between different types of mental health workers, which can provide valuable and indispensable information concerning which profession types are better prepared and more willing to provide FFP and which need further mobilizing and training.

Therefore, our study examined the attitudes, knowledge, skills, and practices in a sample of Chinese mental health workers in respect to FFP, and carried out comparisons between different types of mental health workers in China in terms of FFP. Moreover, we investigated the effects of previous FFP-related experiences, including previous training in FFP, previous involvement in FFP, and previous experiences of caring for sick relatives or friends, on mental health workers’ attitudes, knowledge, skills, and family-focused practices. It was hypothesized that previous FFP-related experiences were positively associated with mental health workers’ attitudes, knowledge, skills, and practices in respect to FFP.

## Methods

### Design

A questionnaire-based, cross-sectional study design was employed to examine the attitudes, knowledge, skills, and practices of a convenience sample of Chinese mental health workers in respect to FFP. This study was approved by the ethics committee of Peking University Sixth Hospital.

### Instruments

The Family-Focused Mental Health Practice Questionnaire (FFMHPQ) is a 53-item, 7-point Likert scale used to investigate the attitudes, knowledge, skills, and practices of mental health workers in respect to FFP. To the best of our knowledge, it is the first and only validated instrument used for measuring mental health workers’ FFP. The FFMHPQ was initially constructed by Maybery et al. in 2012 [28] and its original version contained 49 items that could be further classified into 16 subscales describing diverse aspects of FFP, including family-focused policy and procedure, workplace support for FFP, skill and knowledge, worker confidence in FFP, and actual support to carers and children. Following their initial study [28], Maybery et al. added 4 new items about supporting parenting within the context of mental illness that were also thought important in FFP to the original version of the FFMHPQ, with 2 additional subscales identified (“inter-professional practice” and “supporting parenting within the context of mental illness”) [14, 29]. The participants are asked to rate to what extent they agree or disagree with each item (1 = strongly disagree, 2 = disagree, 3 = slightly disagree, 4 = neither agree or disagree, 5 = slightly agree, 6 = agree, 7 = strongly agree). Subscale scores are calculated as the average of the individual item scores. Higher subscale scores represent more positive appraisals of the corresponding aspects of FFP (e.g., a higher subscale score on “family-focused policy and procedure” represents a more positive appraisal of family-focused policy and procedure in the workplace). The FFMHPQ has sound validity and reliability [28] and has been used with different profession types [27], in different health care settings [30], and in different cultures [14, 31].

In the current study, we translated the FFMHPQ from English to Chinese following the rigorous cross-cultural translation procedure recommended by Beaton & Guillemin [32]. First, two translators with Chinese as their primary language translated the questionnaire independently from English to Chinese. Second, the research team and translators met to compare the two independent translations for consistency, as well as resolving any discrepancies between the two translations in order to develop a combined agreed version. Third, two English first-language translators independently translated the combined version back into English. Last, the research team and translators reviewed all translations and reached consensus on a pre-final version of the translated FFMHPQ for pilot study. Fifteen Chinese mental health workers across different profession types and different workplaces were invited to complete the pre-final version voluntarily. They were then interviewed about the design and layout of the questionnaire, how easy/difficult it was to answer the questions, and what they think were meant by the questions and chosen responses [32]. Based on the pilot study, minor changes were made to the layout and wording of the questionnaire and a final version was developed. The Chinese version of the FFMHPQ had sound face and content validity and the Cronbach’s alpha was 0.932.

Besides the FFMHPQ, a self-constructed questionnaire was administered to the participants, collecting data on participants’ demographics, including age, gender, education, profession type, place of service, and length of service, and previous FFP-related experiences, including previous involvement in FFP, previous training in FFP, and previous experiences of caring for sick relatives or friends.

### Participants and setting

China’s mental health care system is organized into three tiers: community health centers (CHCs), which provide management, treatment, and rehabilitation for community members with mental disorders; secondary psychiatric hospitals (SPHs), which tend to be affiliated with a medium size city, county or district and provide specialist health services for individuals with mental disorders on a regional level; and tertiary psychiatric hospitals (TPHs), which are situated in provincial capitals or major cities and responsible for treating individuals with serious, complex, or uncommon mental health conditions, as well as providing guidance and support to all mental health facilities in the area [33]. There are some other types of facilities that provide mental health care, such as general hospitals and rehabilitation hospitals, but they contribute only a small share to the provision of mental health services in China [34]. In China, there are five main types of mental health workers, i.e., psychiatrists, psychiatric nurses, clinical psychologists, mental health social workers, and occupational therapists. Psychiatrists and psychiatric nurses accounted for over 80 % of China’s mental health workforce [34]. The primary responsibility of psychiatrists is to diagnose and treat mental disorders. Community-based mental health services are provided by a cadre of psychiatrists who are also responsible for the management of community members with mental disorders. Psychiatric nurses usually provide nursing care to inpatients in psychiatric hospitals. Clinical psychologists provide counseling and psychotherapy to individuals with mental disorders as well as their families. Mental health social workers assess social needs of patients and families, provide counseling, and refer them to community resources to assist in recovery of mental illness. Occupational therapists work with patients with mental disabilities and their families to help patients develop, improve, and maintain their skills needed for daily living and working.

In January 2020, the questionnaires were administered to a convenience sample of mental health workers in Beijing, China. They were enrolled from one TPH, two SPHs, and all the CHCs situated in three districts of Beijing. The inclusion criteria included: (1) being full-time staff; (2) working with adult patients with mental illness; (3) having worked for more than six months as mental health workers. The exclusion criteria included: (1) being visiting fellows, students, or part-time staff; (2) working exclusively with children or adolescents with mental illness. Based on previous studies [14, 28], a sample size of *n* = 300 was needed to quantify a difference on mean subscale scores between two different types of mental health workers at a confidence level of 0.05 with 90 % power.

### Data collection

For the TPH and SPHs, the anonymous, online questionnaires were first sent to the hospital directors who assisted us to distribute the questionnaires to all their eligible staff (*n* = 120 for the TPH and *n* = 306 for the SPHs) via the WeChat-based survey platform “Questionnaire Star”. For the CHCs, the questionnaires were sent through the district mental health officers who took charge of the community mental health services in the local districts to all the eligible community mental health workers (*n* = 205) via the “Questionnaire Star”. To increase the response rate, several information sessions were organized to introduce the aims and objectives of our study, as well as other concerns held by the potential participants. Our contact details were also attached with the questionnaires so that whoever required any clarification or further information about our study could contact us. The questionnaires were distributed via WeChat once per week for three consecutive weeks so that more mental health workers could notice our survey. To encourage survey completion, participants who completed the questionnaires were rewarded with a random amount of cash (anywhere from 1 Chinese yuan to 50 Chinese yuan). In addition, one captcha question was added at the end of the questionnaire in order to prevent bots or other automated software from answering it.

### Data analysis

Continuous variables are presented as means and standard deviations (SD) or medians and interquartile ranges (IQR) as appropriate, and categorical variables as counts and percentages. One-way ANOVA tests with Scheffe’s post-hoc method or Kruskal-Wallis tests with Dunn-Bonferroni post-hoc method were applied for multiple comparisons of continuous variables as appropriate. Pearson’s chi-squared tests or Fisher’s exact tests with Bonferroni post-hoc method were applied for multiple comparisons of categorical variables. Multivariate linear regression analyses were undertaken to identify the effects of previous FFP-related experiences on FFMHPQ subscale scores after adjusting for other variables. The significance level was 0.05. All statistical analyses were conducted with SPSS software version 24.0 (Chicago, IL, USA).

## Results

### Demographics, occupational information, and previous FFP-related experiences

In total, 515 mental health workers completed the questionnaire, yielding a response rate of 82 %. Table 1 shows the demographics, occupational information, and previous FFP-related experiences of our sample, as well as the results of multiple comparisons across different profession types. Most participants in our study were either psychiatrists (213 [41.4 %]) or psychiatric nurses (269 [52.5 %]). Other profession types of mental health workers were in relatively small numbers (clinical psychologists: 20 [3.9 %]; mental health social workers: 9 [1.7 %]; occupational therapists: 4 [0.8 %]). In order to achieve adequate statistical power in our study, these three types were combined into one group, i.e. allied mental health professionals (AMHPs), during analysis. In our sample, 174 of 515 (33.8 %) participants provided services in the CHCs, 251 (48.7 %) in the SPHs, and 90 (17.5 %) in the TPH. 374 of 515 (72.6 %) participants were female. A greater percentage of females were found in psychiatric nurses (214 of 269 [79.6 %]) than in psychiatrists (133 of 213 [62.4 %]). The median (IQR) age of psychiatric nurses in our sample was 32 (26.5–36) years, smaller than that of psychiatrists (36 [31.5–45]) and AMHPs (39 [32.5–44]). Most participants held a bachelor’s degree (281 of 515 [54.6 %]) or an associate degree (182 of 515 [35.3 %]). Only 3 of 269 (1.1 %) psychiatric nurses holding a postgraduate degree, whereas 42 of 213 (19.8 %) psychiatrists and 7 of 33 (21.2 %) AMHPs held a postgraduate degree. The median (IQR) length of service was 8 (3–13) years. In our sample, 212 (41.2 %) participants reported previous training in FFP, and 159 (30.9 %) reported previous involvement in FFP. There were a smaller percentage of psychiatric nurses (53 of 269 [19.7 %]) that had ever been involved in FFP than psychiatrists (90 of 213 [42.3 %]) and AMHPs (16 of 33 [48.5 %]). Previous experiences of caring for sick relatives or friends were found in 300 (58.3 %) participants.

**Table 1:** Demographics, occupational information, and previous FFP-related experiences of the participants

Characteristics, median (IQR) or N (%)	All (*n* = 515)	Profession types			Post-hoc tests
		Psychiatrists (*n* = 213)	Psychiatric nurses (*n* = 269)	AMHPs (*n* = 33)	
Age, years	34 (29–40)	36 (31.5–45)***	32 (26.5–36)***	39 (32.5–44)***	2 < 1, 2 < 3
Gender					
Female	374 (72.6)	133 (62.4)***	214 (79.6)***	27 (81.8)***	1 < 2
Male	141 (27.4)	80 (37.6)***	55 (20.4)***	6 (18.2)***	2 < 1
Education					
Associate degree	182 (35.3)	40 (18.8)***	135 (50.2)***	7 (21.2)***	1 < 2, 3 < 2
Bachelor’s degree	281 (54.6)	131 (61.5)***	131 (48.7)***	19 (57.6)***	2 < 1
Postgraduate degree	52 (10.1)	42 (19.8)***	3 (1.1)***	7 (21.2)***	2 < 1, 2 < 3
Place of service					
CHC	174 (33.8)	108 (50.7)***	44 (16.4)***	22 (66.7)***	2 < 1, 2 < 3
SPH	251 (48.7)	56 (26.3)***	189 (70.3)***	6 (18.2)***	1 < 2, 3 < 2
TPH	90 (17.5)	49 (23.0)***	36 (13.4)***	5 (15.2)***	2 < 1
Length of service, years	8 (3–13)	10 (4–14)*	7 (3–12)*	10 (4–20)*	2 < 1
Previous training in FFP	212 (41.8)	112 (52.6)***	75 (27.9)***	25 (75.8)***	2 < 1 < 3
Previous involvement in FFP	159 (30.9)	90 (42.3)***	53 (19.7)***	16 (48.5)***	2 < 1, 2 < 3
Previous experiences of caring for sick relatives or friends	300 (58.3)	117 (55.0)	163 (60.6)	20 (60.7)	-

*Abbreviations:* *AMHP* allied mental health professional; *CHC* community health center; *SPH* secondary psychiatric hospital; *TPH* tertiary psychiatric hospital; *FFP* family-focused practice; *IQR* interquartile range

Notes: * *P* < 0.05; ** *P* < 0.01; *** *P* < 0.001

### Attitudes, knowledge, skills, and practices across different profession types

As shown in Table 2, the lowest mean subscale scores were given to “location issues in relation to FFP” (3.23, SD 1.21) and “service availability” (3.37, SD 1.25), which indicated a lack of family-focused mental health services in the locality. The highest mean subscale score was given to “the need for further training” (5.40, SD 1.07), which suggested that most participants in our study considered it necessary to undertake further training in FFP. Psychiatric nurses reported less workload than psychiatrists (3.95, SD 0.92 vs. 3.51, SD 1.15; *P* < 0.001). Psychiatric nurses provided less support to families and children than psychiatrists (4.27, SD 1.21 vs. 4.65, SD 1.16; *P* = 0.003) and AMHPs (4.27. SD 1.21 vs. 4.84, SD 1.03; *P* = 0.035). In addition, psychiatric nurses were less willing to receive future training in FFP than psychiatrists (5.21, SD 1.16 vs. 5.58, SD 0.94; *P* = 0.001) and AMHPs (5.21, SD 1.16 vs. 5.80, SD 0.86; *P* = 0.011). Psychiatric nurses were more likely to think that there was no opportunity for engagement with families than psychiatrists (3.44, SD 0.98 vs. 3.73, SD 1.11; *P* = 0.009) and AMHPs (3.44, SD 0.98 vs. 3.98, SD 0.80; *P* = 0.018). We found no significant differences on skill and knowledge (*F* = 0.629, *P* = 0.534) and confidence (*F* = 2.805, *P* = 0.061) across different profession types. Moreover, we noticed that AMHPs scored higher than psychiatrists on most subscales, including “skill and knowledge”, “confidence”, “support to families and children”, and “the need for future training”. However, no differences between AMHPs and psychiatrists were statistically significant.

**Table 2:** Means and standard deviations of FFMHPQ subscale scores in different profession types of participants

Subscale scores^a^	All (*n* = 515)	Profession types			Post-hoc tests
		Psychiatrists (*n* = 213)	Psychiatric nurses (*n* = 269)	AMHPs (*n* = 33)	
Workplace support for family-focused practice	4.74 (1.36)	4.59 (1.46)	4.85 (1.28)	4.71 (1.33)	-
Location issues in relation to family-focused practice	3.24 (1.21)	3.11 (1.26)*	3.39 (1.17)*	3.00 (1.20)*	1 < 2
Time and workload	3.75 (1.05)	3.51 (1.15)***	3.95 (0.92)***	3.69 (1.14)***	1 < 2
Family-focused policy and procedure	4.40 (1.33)	4.06 (1.30)***	4.78 (1.23)***	3.59 (1.38)***	1 < 2, 3 < 2
Family-focused professional development opportunities	4.43 (1.33)	4.47 (1.26)	4.38 (1.40)	4.52 (1.25)	-
Coworker support	4.72 (1.16)	4.62 (1.11)	4.82 (1.16)	4.52 (1.39)	-
Providing support to the family and client for parenting	4.81 (0.96)	4.89 (0.89)*	4.72 (1.03)*	5.07 (0.74)*	-
Worker confidence in family-focused practice	3.93 (1.30)	3.78 (1.34)	4.01 (1.28)	4.23 (1.22)	-
Providing support to families and children	4.46 (1.19)	4.65 (1.16)***	4.27 (1.21)***	4.84 (1.03)***	2 < 1, 2 < 3
Engaging with family	3.59 (1.04)	3.73 (1.11)**	3.44 (0.98)**	3.98 (0.80)**	2 < 1, 2 < 3
Assessing impact of parental mental illness on the child	4.46 (1.16)	4.53 (1.22)	4.40 (1.12)	4.53 (1.10)	-
The need for further training	5.40 (1.07)	5.58 (0.94)***	5.21 (1.16)***	5.80 (0.86)***	2 < 1, 2 < 3
Skill and knowledge	4.29 (0.78)	4.27 (0.90)	4.29 (0.65)	4.44 (0.86)	-
Service availability	3.37 (1.25)	3.21 (1.32)**	3.55 (1.17)**	2.91 (1.16)**	1 < 2, 3 < 2
Promoting family connectedness	4.29 (0.80)	4.33 (0.87)	4.24 (0.73)	4.48 (0.82)	-
Referrals for family members	4.29 (0.93)	4.36 (1.04)	4.20 (0.81)	4.50 (1.05)	-
Inter-professional practice	5.36 (1.03)	5.56 (0.89)***	5.17 (1.10)***	5.63 (0.97)***	2 < 1, 2 < 3
Supporting parenting within the context of mental illness	3.91 (1.03)	3.90 (1.09)	3.88 (0.98)	4.28 (0.97)	-

a. A subscale score of < 4, 4, and > 4 indicates a negative, neutral, and positive appraisal respectively

*Abbreviations:* *AMHP* allied mental health professional; *FFMHPQ* Family-Focused Mental Health Practice Questionnaire

Notes: * *P* < 0.05; ** *P* < 0.01; *** *P* < 0.001

### Effects of previous FFP-related experiences

Table 3 shows the effects of previous FFP-related experiences on FFP. In order to separate out the effects of previous FFP-related experiences from other variables, multivariate linear regression analyses were performed. It was found that previous training in FFP was positively associated with more confidence (*B* = 0.349, *P* = 0.022), more knowledge and skills about working with patients and their children (*B* = 0.206, *P* = 0.021) and supporting parenting within the context of mental illness (*B* = 0.459, *P* < 0.001), after adjusting for age, gender, education, profession type, place of service, length of service, previous involvement in FFP, and previous experiences of caring for sick relatives or friends. Mental health workers who had previously been involved in FFP showed more knowledge and skills about working with patients and their children (*B* = 0.258, *P* = 0.012) and supporting parenting within the context of mental illness (*B* = 0.308, *P* = 0.023). However, we found no associations between previous training or involvement in FFP and mental health workers’ practical support to their patients’ family members. In contrast, those who had previously cared for sick relatives or friends showed more support to patients’ family members (*B* = 0.249, *P* = 0.018), and were more willing to undertake further training in FFP (*B* = 0.217, *P* = 0.024).

**Table 3:** Means and standard deviations of FFMHPQ subscale scores in participants with or without previous FFP-related experiences

Subscale scores^a^	Previous training in FFP^b^		Previous involvement in FFP^c^		Previous experiences of caring for sick relatives or friends^d^	
	Yes (*n* = 212)	No (*n* = 303)	Yes (*n* = 159)	No (*n* = 356)	Yes (*n* = 300)	No (*n* = 215)
Workplace support for family-focused practice	4.81 (1.35)	4.68 (1.37)	4.79 (1.43)	4.71 (1.34)	4.77 (1.42)	4.70 (1.27)
Location issues in relation to family-focused practice	3.32 (1.28)	3.21 (1.16)	3.41 (1.36)*	3.18 (1.14)*	3.35 (1.29)	3.10 (1.09)
Time and workload	3.89 (1.13)*	3.66 (0.98)*	3.88 (1.18)	3.70 (0.98)	3.80 (1.13)	3.68 (0.93)
Family-focused policy and procedure	4.27 (1.33)	4.50 (1.32)	4.25 (1.35)	4.47 (1.31)	4.44 (1.34)	4.35 (1.32)
Family-focused professional development opportunities	4.59 (1.28)	4.31 (1.36)	4.63 (1.25)	4.34 (1.36)	4.53 (1.34)*	4.28 (1.31)*
Coworker support	4.76 (1.16)	4.68 (1.15)	4.73 (1.20)	4.71 (1.14)	4.78 (1.22)	4.63 (1.06)
Providing support to the family and client for parenting	4.99 (0.95)	4.69 (0.95)	5.00 (1.00)	4.73 (0.93)	4.84 (1.01)	4.77 (0.89)
Worker confidence in family-focused practice	4.19 (1.32)*	3.75 (1.26)*	4.19 (1.34)	3.82 (1.27)	4.03 (1.34)	3.80 (1.25)
Providing support to families and children	4.78 (1.18)	4.24 (1.15)	4.83 (1.17)	4.30 (1.17)	4.59 (1.17)*	4.29 (1.20)*
Engaging with family	3.78 (1.08)*	3.46 (0.99)*	3.74 (1.09)	3.53 (1.01)	3.59 (1.06)	3.59 (1.01)
Assessing impact of parental mental illness on the child	4.54 (1.15)	4.40 (1.17)	4.54 (1.20)	4.42 (1.15)	4.47 (1.20)	4.44 (1.11)
The need for further training	5.46 (1.04)	5.35 (1.09)	5.44 (1.11)	5.38 (1.06)	5.48 (1.09)*	5.29 (1.04)*
Skill and knowledge	4.47 (0.80)*	4.17 (0.74)*	4.49 (0.83)*	4.21 (0.73)*	4.35 (0.83)	4.21 (0.69)
Service availability	3.41 (1.29)	3.34 (1.22)	3.43 (1.37)	3.35 (1.19)	3.43 (1.30)	3.29 (1.17)
Promoting family connectedness	4.44 (0.82)	4.19 (0.77)	4.42 (0.90)	4.23 (0.75)	4.36 (0.80)	4.20 (0.79)
Referrals for family members	4.55 (0.95)***	4.10 (0.87)***	4.49 (1.02)	4.19 (0.87)	4.31 (0.95)	4.25 (0.90)
Inter-professional practice	5.42 (1.02)	4.23 (1.01)	5.41 (1.08)	5.33 (1.00)	5.44 (1.04)*	5.25 (0.99)*
Supporting parenting within the context of mental illness	4.24 (1.01)***	3.69 (0.98)***	4.22 (1.11)*	3.78 (0.96)*	3.98 (1.08)	3.82 (0.95)

a. A subscale score of < 4, 4, and > 4 indicates a negative, neutral, and positive appraisal respectively

b. All the results were adjusted for age, gender, education, profession type, place of service, length of service, previous involvement in FFP, and previous experiences of caring for sick relatives or friends

c. All the results were adjusted for age, gender, education, profession type, place of service, length of service, previous training in FFP, and previous experiences of caring for sick relatives or friends

d. All the results were adjusted for age, gender, education, profession type, place of service, length of service, previous training in FFP, and previous involvement in FFP

*Abbreviations:* *FFMHPQ* Family-Focused Mental Health Practice Questionnaire; *FFP* family-focused practice

Notes: * *P* < 0.05; ** *P* < 0.01; *** *P* < 0.001

## Discussion

This was the first study that investigated Chinese mental health workers’ attitudes, knowledge, skills, and practices in respect to FFP and, to the best of our knowledge, was the largest one of its kind in the world. Our study included a relatively large number of psychiatrists (*n* = 214), larger than any previous study investigating FFP in mental health workers in other countries (e.g., Thailand [14], Australia [27]). Therefore, it can act as a benchmark for future research about FFP in psychiatrists.

Our study found that psychiatrists in China provided more support for carers and children of patients with mental illness than psychiatric nurses. This finding is in line with Tungpunkom et al.’s survey in Thai mental health workers [14]. However, our study observed no significant differences on workers’ knowledge, skills, and confidence about FFP between psychiatric nurses and psychiatrists. In fact, psychiatrists in our study scored much less on knowledge, skills, and confidence about FFP compared to their Thai counterparts, whereas psychiatric nurses in these two countries scored equivalent to each other [14]. More detailed investigations are needed to help explain this cross-cultural discrepancy. In China, due to a severe shortage of clinical psychologists, mental health social workers, and occupational therapists, many of their responsibilities lie with psychiatrists, making psychiatrists in China often faced with heavy workloads. In our study, psychiatrists reported less spare time to care for families and children of patients with mental illness than psychiatric nurses. However, it was also found that psychiatrists in China were more willing to receive further training in FFP than psychiatric nurses. There may be several explanations to this finding. First, most psychiatric nurses in China provide care for inpatients in locked psychiatric wards where there are few opportunities for them to communicate with patients’ families and children. Second, the main duties of psychiatric nurses in China are to follow psychiatrists’ written orders, to administer treatment protocols, and to providing nursing care for inpatients. On the contrary, psychiatrists in China often have a responsibility to advise and support their patients’ families and children. Also, in Chinese culture, family members are often more willing and accustomed to consult psychiatrists who they regard are more knowledgeable and trustable than other mental health workers. Therefore, psychiatrists in China may be more aware of the needs of families and children of patients with mental illness, thus showing more motivations to increase their knowledge and skills on FFP. This finding is particularly important because it suggests that engaging psychiatrists in the future work of dissemination and implementation of FFP might be an appropriate option, given their stronger motivations on FFP. However, on the other hand, psychiatric nurses are the largest group in China’s mental health workforce and they also admitted more time to provide family-focused services than psychiatrists. Therefore, it is also reasonable to make measures to mobilize more psychiatric nurses to engage in family-focused mental health services. For this purpose, the scope of psychiatric nursing practice in China must be broadened to incorporate the role of addressing the needs of patients’ families and children. More opportunities to engage with families and children should be created for psychiatric nurses. For instance, when working in psychiatric wards, psychiatrists should make every effort to involve psychiatric nurses in family meetings for patients with mental illness and collaborate with psychiatric nurses on the planning of support for their patients’ families and children.

In China, psychiatrists and psychiatric nurses account for over 80 % of mental health workers, whereas clinical psychologists, mental health social workers, and occupational therapists are desperately scarce [34]. Therefore, we did not enroll a considerable number of clinical psychologists, mental health social workers, and occupational therapists in our study, and chose to combine them into one group, i.e., AMHPs, during analysis. We observed that AMHPs scored the highest among different profession types on most aspects of FFP. In particular, they showed more knowledge and skills about working with families and children of patients with mental illness than psychiatrists and psychiatric nurses. They also felt more confident of providing family-focused mental health services and offered more support to family carers and children. Moreover, they expressed more interest in future training in FFP. These findings tally with previous research in Australia [27], Thailand [14], and Ireland [35]. In Australia, it was found that psychologists and social workers performed better than psychiatric nurses on almost all variables of the FFMHPQ [27]. In Thailand, mental health social workers also scored higher than other profession types on confidence, practical support, and willingness to receive training in FFP [14]. This may be because social workers and psychologists are more often exposed to family-focused perspectives during education and professional development. Also, mental health social workers, clinical psychologists, and occupational therapists in China more often engage families in recovery of people with mental illness than psychiatrists and psychiatric nurses in daily work.

We also examined the effects of previous experiences related to FFP on mental health workers’ attitudes, knowledge, skills, and family-focused practices. Our results indicated that previous training in FFP had a positive effect on mental health workers’ knowledge, skills, and confidence about FFP. However, previous training in FFP was not associated with more support to families and children. It is plausible that there exist some constraints for trained mental health workers to provide family-focused services to carers and children of patients. These constraints may include lack of motivation, heavy workload, no opportunities to receive supervision, and lack of support from colleagues and institutions [36]. In Portugal, one study evaluated the effectiveness of a training program on FFP and found that, although the training program could effectively increase mental health workers’ knowledge and skills on FFP, it did not promote workers’ actual support to carers and children in their clinical work [31]. Therefore, in order to enhance family-focused mental health services, multilevel strategies are needed to address various factors that may impede the effective implementation of family-focused services. For example, ongoing supervision and support on FFP should be provided to trained mental health workers. Mental health care facilities should encourage and incentivize mental health workers to engage in the provision of family-focused services. In addition, family-focused policies and guidelines should be enacted at national and regional level to promote family-focused mental health care.

Our study had several limitations. First, because of a shortage of clinical psychologists, mental health social workers, and occupational therapists in China, the sample sizes of these three profession types in our study were relatively small. In order to achieve adequate statistical power, one compromise that we made was to combine them into one analysis group, i.e., AMHPs. However, it also made it impossible to examine the attitudes, knowledge, skills, and practices in respect to FFP among each of these three profession types. Second, our sample was not a nationwide representative sample of mental health workers in China. However, the majority of mental health workers in China are working in large cities located along the eastern coast of China, and Beijing has the largest number of mental health workers per 100,000 population among these large cities [34]. Therefore, our study can still provide meaningful information concerning Chinese mental health workers’ attitudes, knowledge, skills, and family-focused practices. Third, our study used a cross-sectional survey method which did not allow assessment of the direction of effect for the associations described above.

## Conclusions

Our study showed that psychiatrists in China provided more support to families and children of patients with mental illness and were more willing to receive further training in FFP than psychiatric nurses. As the provision of family-focused mental health services to COPMI has been written into the newly released *China National Action Plan for Child and Adolescent Mental Health* [20], engaging psychiatrists in the future work of dissemination and implementation of FFP might be a solid start for China. It is also worth expecting that clinical psychologists, mental health social workers, and occupational therapists can also be highly motivated to perform FFP in the future. In addition, our study implied that training in FFP itself didn’t necessarily associate with more support to carers and children in clinical practices. Studies are needed to evaluate various determinants that may influence the adoption of FFP among Chinese mental health workers, based on which multilevel strategies need developing to scale up family-focused mental health services in China.

## Data Availability

Additional data available from the corresponding author at guanlili@bjmu.edu.cn.

## Abbreviations

AMHP: allied mental health professional
CHC: community health center
COPMI: children of parents with mental illness
FFMHPQ: Family-Focused Mental Health Practice Questionnaire
FFP: family-focused practices
IQR: interquartile range
SD: standard deviation
SPH: secondary psychiatric hospital
TPH: tertiary psychiatric hospital
